# Editorial: Early Domestication and Artificial Selection of Animals

**DOI:** 10.3389/fgene.2022.841252

**Published:** 2022-02-25

**Authors:** Hai Xiang, Martijn F. L. Derks, Guoqiang Yi, Xingbo Zhao

**Affiliations:** ^1^ Guangdong Provincial Key Laboratory of Animal Molecular Design and Precise Breeding, School of Life Science and Engineering, Foshan University, Foshan, China; ^2^ Animal Breeding and Genomics, Wageningen University and Research, Wageningen, Netherlands; ^3^ Shenzhen Branch, Guangdong Laboratory of Lingnan Modern Agriculture, Genome Analysis Laboratory of the Ministry of Agriculture and Rural Affairs, Agricultural Genomics Institute at Shenzhen, Chinese Academy of Agricultural Sciences, Shenzhen, China; ^4^ National Engineering Laboratory for Animal Breeding, Key Laboratory of Animal Genetics, Breeding and Reproduction, Ministry of Agriculture and Rural Affairs, College of Animal Science and Technology, China Agricultural University, Beijing, China

**Keywords:** animal, domestication, artificial selection, breed formation, adaptive evolution

The domestication of several key farm animals during the early Holocene initiated the transition from the hunter-gatherer lifestyle to food production. Early domestication started by the recognition of biological characteristics in animals that would benefit human demands. Since their initial domestication, animals have been translocated worldwide and selected intensively, which has resulted in substantial genomic adaption of various traits such as tameness, growth, reproduction, etc.

Beyond the general interest in animal domestication, numerous important genetic loci selected by humans have been detected. These researches contribute to a better understanding of early domestication and subsequent artificial selection. However, the primary selective signals of domestication have been lost by migration and introgression of domesticated and wild animals.

In this research topic we cover genetic signals associated with early domestication. Moreover, we cover several aspects of human driven selection basis of the phenotypic differentiation and environmental adaptation and the effect of artificial selection on domestic animals.

## Early Domestication

Using paleo genomic technologies, Wang et al. and Zhang et al. retrieved ancient genetic information to unravel the spatial and temporal histories of the appearance and spread of domesticated animals. By analyzing three near-complete mitochondrial genomes of donkey specimens (2,350–300 cal. BP), Wang et al. revealed that two maternal donkey lineages had been introduced into Midwestern China at least at the opening of Silk Road (approximately the first century BC). The further analyses confirmed that the two donkey lineages experienced somewhat different past demographic expansion histories, and donkeys were supposed to have undergone at least two independent domestication events. Meanwhile, Zhang et al. obtained three near-complete mitochondrial genomes from bovine remains dating back cal. 4,000 years in North China. For the first time at the mitogenome level, phylogenetic analyses confirmed the ∼4,000-year-old bovines from North China as taurine cattle, which originated from the Near East. Ancient Chinese cattle had a genetic contribution to the modern taurine cattle of South China. Furthermore, a rapid decrease in the female effective population size cal. 4,650 years ago and a steep increase cal. 1,990 years ago occurred in Chinese taurine cattle.

## Genetic Diversity and Introgression

After the early domestication, contiguous breeding had been conducted on animals, leading to distinct genetic diversity between breeds or subspecies. By refining the genetic map of chicken MHC B-F/B-L region, Yuan et al. illustrated that Chinese domestic chicken breeds showed higher genetic diversity among 21 populations, suggesting their broad-spectrum resistance to pathogens. The highest and lowest genetic diversity of the MHC B-F/B-L region occurred in the indigenous chicken breeds and highly inbred line chickens, respectively, which might result from the long-term intense selection for decades.

Along with human-mediated dispersal and translocation, genetic introgression extensively happened between subspecies of domestic animals from different origins. Moreover, crossbreeding was often conducted to breed for specific traits of interest or to improve livestock’s productivity. Wang et al. identified significant human-mediated introgression from European pigs into the genome of Chinese indigenous pigs. The introgression derived from Duroc pigs was captured for Henan indigenous pigs, especially in Nanyang black pigs. The introgressive *NR6A1*, *GPD2*, and *CSRNP3* genes were suggested to contribute to pig body size and feed conversion ratio, providing evidence for the effect of introgression and selection on reshaping the pig genome.

Finally, the review by Derks and Steensma explored a range of causative variants under balancing selection including loss-of-function variation and regulatory variation in domestic animals. This paper highlights some of the negative consequences associated with strong artificial selection. The authors marked various deleterious variants under balancing selection that had been discovered in various livestock species including pig (*Sus scrofa*), cow (*Bos taurus*), sheep (*Ovis aries*), chicken (*Gallus gallus*) and the horse (*Equus caballus*). The functional consequences at the molecular, phenotypic, and population level were described and summarized to provide a resource for further study.

## Signatures of (Artificial) Selection

Strong artificial selection has shaped the genome of domestic species. Given that animals had been subjected to artificial selection and improvement of various phenotypes with distinct purposes, great changes in production performance and adaptability have occurred in domestic populations. Li et al. performed genomic analyses on the genetic difference and growth traits in American and Canadian Duroc lines. The results showed apparent genetic differentiation between the two Duroc lines, and showed multiple selective regions and candidate genes associated with growth traits. By making phenotypic gradient differential population pairs, Shen et al. detected a total of 427 and 307 trait-specific selection signatures for meat quality in Large White pigs through different analyses. Several overlapping genes within the trait-specific selection signatures were responsible for the phenotypes including fat metabolism and muscle development.

## Transcriptomic and Epigenomic Response to Selection

With the aim to understand the genetic basis for adaptation and plasticity of contrasted climates in small ruminants, Denoyelle et al. characterized both epigenetic and genetic variations by contrasting 22 sheep and 21 goats from both sides of a climate gradient. For both species, a series of candidate genes in relation to environmental perception, immunity, reproduction and production were detected under selection with high vs. low temperature annual variations. Two differentially methylated genes, namely *AGPTA4* in goat and *SLIT3* in sheep were identified and are both related to milk production and muscle development. This work provided an explorative example to investigate the transcriptional regulatory elements and epigenetic modifications driving adaptations and selection of domesticated animals. On the other hand, Chen et al. tested the different phenotypic plasticity between slow- and fast-growing chicken in response to feed restriction. The results showed that feed restriction profoundly affected on the brain organ index and plasma dopamine in the slow- and fast-growing chickens. And the transcriptome profile analysis suggested that feed restriction might result in issues relating to cardiovascular and neurodegenerative diseases in the fast-growing breed tested, but not in the slow-growing breed.

In summary, this Research Topic collected extensive genetic evidence related to early and late domestication and in relation to current day artificial selection.

